# Immunity Promotes Virulence Evolution in a Malaria Model

**DOI:** 10.1371/journal.pbio.0020230

**Published:** 2004-06-22

**Authors:** Margaret J Mackinnon, Andrew F Read

**Affiliations:** **1**School of Biological Sciences, University of EdinburghEdinburghUnited Kingdom

## Abstract

Evolutionary models predict that host immunity will shape the evolution of parasite virulence. While some assumptions of these models have been tested, the actual evolutionary outcome of immune selection on virulence has not. Using the mouse malaria model, *Plasmodium chabaudi,* we experimentally tested whether immune pressure promotes the evolution of more virulent pathogens by evolving parasite lines in immunized and nonimmunized (“naïve”) mice using serial passage. We found that parasite lines evolved in immunized mice became more virulent to both naïve and immune mice than lines evolved in naïve mice. When these evolved lines were transmitted through mosquitoes, there was a general reduction in virulence across all lines. However, the immune-selected lines remained more virulent to naïve mice than the naïve-selected lines, though not to immunized mice. Thus, immune selection accelerated the rate of virulence evolution, rendering parasites more dangerous to naïve hosts. These results argue for further consideration of the evolutionary consequences for pathogen virulence of vaccination.

## Abstract 2

Immunisation against malaria can promote the evolution of more virulent pathogens in mice, making non-immunised mice more vulnerable -- a result with important implications for the development of malaria vaccines

## Introduction

Genetic variation in pathogen virulence (harm to the host) has been found whenever it has been looked for. A considerable body of theory, based on the transmission consequences of virulence, has been developed to predict how natural selection will act on this genetic variation and how it will shape virulence levels in natural populations of disease-causing organisms (Frank 1996; Dieckmann et al. 2002). For instance, natural or vaccine-acquired host immunity protects hosts from dying, thereby relieving the parasite of the potential fitness costs of prematurely shortened infections. Thus, host populations with high levels of immunity can maintain more virulent pathogens than can naïve host populations (Gandon et al. 2001). To date, the best example of virulence evolving upwards in response to enhanced levels of host defense comes from an uncontrolled “experiment” in the field: upon release into a highly susceptible host population, the myxomatosis virus evolved lower virulence (Fenner and Ratcliffe 1965) but then later increased in virulence once the host population had evolved resistance (Best and Kerr 2000).

As well as altering between-host selection pressures on virulence, host immunity can alter the nature of inhost selection. Different directions of virulence evolution are expected depending on the details of inhost competition among parasites (e.g., Nowak and May 1994; Van Baalen and Sabelis 1995; Chao et al. 2000; Brown et al. 2002). Unfortunately, these details are not well understood for any pathogen (Read and Taylor 2001). The only generality is that serial passage of pathogens almost always increases virulence (Ebert 1998), implying that virulent variants have a fitness advantage within hosts. However, all serial passage experiments of which we are aware were conducted in immunologically naïve hosts, so the effects of immunity on virulence evolution are unknown. In theory, immunity could impose selection in several ways. For instance, lower parasite loads should reduce resource competition (e.g., for red blood cells) among parasites occupying the same host, but increase the competition for enemy-free space (e.g., by immune evasion). This could lead to more aggressive parasites racing to stay ahead of proliferating immune responses (Antia et al. 1994); it could also lead to the evolution of novel antigenic variants that have a selective advantage only in immunized hosts. Immunization will also alter the timing of immune selection, thus potentially selecting for changes in parasite life history parameters that affect virulence, such as an earlier or higher rate of production of transmission stages (Koella and Antia 1995). Finally, the rate at which virulence evolution occurs may be limited by the size of the parasite population inside the host, and therefore may be retarded by host immunity. Thus, at least in theory, there are many potential consequences for virulence evolution of prior host immunity, both long-term and short-term in nature.

One barrier to testing theoretical models of virulence evolution is that the models typically predict the outcome at evolutionary and epidemiological equilibrium. New equi-libria may or may not take a long time to reach, but will in any case depend on the dynamics of the host population and the environmental conditions under which transmission occurs: this means that experimental evolution to new equilibria will be hard to study in the laboratory for medically relevant pathogens. However, the short-term consequences for virulence evolution, which are at least as important to public health policy as the long-term consequences, may be more tractable. This is especially true for diseases for which animal models are available.

In this study, we begin the empirical effort to determine the likely direction of immune-mediated virulence evolution by performing experimental evolution of the rodent malaria parasite, Plasmodium chabaudi, in laboratory mice. We evolved multiple lines of P. chabaudi in immunized and naïve mice by repeated serial passage of blood-stage parasites (i.e., bypassing the normally obligate mosquito vector) starting from two different starting populations. After 20 passages, the lines had evolved sufficiently to make comparisons between the immune-selected lines (I-lines) and naïve-selected lines (N-lines) for virulence and transmissibility.

## Results/Discussion

**Figure 1:**
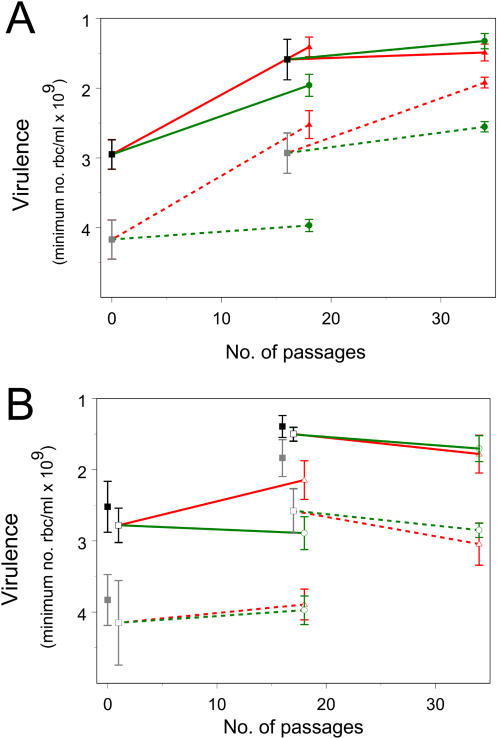
Virulence Evolution in Mouse Malaria during Serial Passage in Immunized Versus Naïve Mice Virulence was measured by minimum red blood cell density (y-axis) in lines of P. chabaudi before (“ancestral lines;”black and gray symbols) and after serial passage through immunized (“I-lines;” red lines and triangles) or naïve (“N-lines,” green lines and circles) mice before (A) and after (B) mosquito transmission. Evolved and ancestral lines were compared in both naïve (solid lines) and immunized mice (broken lines). Filled symbols, before mosquito transmission; open symbols, after mosquito transmission. Lines were selected from an avirulent, “unadapted” clone (CW-0; left set of lines) and a virulent, “preadapted” ancestral population (CW-A; right): the latter was derived from the former by 12 serial passages in a previous experiment (Mackinnon and Read 1999b). Each symbol (with ± 1 standard error based on the variance between subline means) represents the mean of mice infected with an ancestral line or a set of passaged lines (i.e., five sublines, two mice per subline). Prior to mosquito transmission (A), differences between the I-lines and N-lines were significant in three out of the four cases (*p* < 0.05 for lines from the unadapted line infecting naïve mice, *p* < 0.01 for unadapted infecting immunized, and *p* < 0.001 for preadapted infecting immunized): in the fourth case (*p* > 0.1 for preadapted infecting naïve), virulence of the ancestral line was already apparently near-maximal. After mosquito transmission (B), the differences between the I-lines and N-lines remained the same in naïve mice as before transmission (interaction between the mosquito transmission effect and the I-line-versus-N-line difference was *p* > 0.7 in both the unadapted and preadapted cases). However, these line differences were eliminated in immunized mice (interaction term: *p* = 0.02 for the unadapted case, *p* = 0.08 for the preadapted case). Mosquito transmission significantly reduced the virulence of the preadapted ancestral line in immunized mice (*p* = 0.03) but not in the other ancestral-line-by-immune-treatment combinations (*p* > 0.2 in these cases). In the selection lines, mosquito transmission significantly reduced the virulence in five out of the eight comparisons (*p* = 0.009 and *p* = 0.13 in the N-lines in naïve mice derived from CW-0 and CW-A, respectively, with values of *p* = 0.55 and *p* = 0.005 in N-lines in immunized mice, *p* = 0.022 and *p* = 0.26 in I-lines in naïve mice, and *p* = 0.006 and *p* < 0.0001 in I-lines in immunized mice). Ancestral pretransmission lines had similar levels of virulence in the separate pretransmission and posttransmission experiments, with the exception of the preadapted ancestral line in immunized mice, which had higher virulence in the latter than the former (*p* = 0.002). Similar results to the above were obtained when virulence was measured by maximum weight loss (unpublished data). No deaths occurred during the pretransmission experiments, but in addition to the one death that occurred early in the infection prior to the occurrence of any weight loss or anemia (excluded from analyses), five occurred in the posttransmission experiment, four of these in naïve mice (two in the N-lines, one in the I-lines, and one in the nontransmitted ancestral line, all derived from the preadapted line) and one in an immunized mouse (preadapted, nontransmitted ancestral line).

We found that both the I-lines and N-lines evolved to become more virulent than their ancestral populations, but the I-lines became even more virulent than the N-lines (Figure 1A). This higher virulence was manifest in both naïve and immunized mice. When the lines were transmitted through mosquitoes, there was generally a reduction in virulence across all the lines, but the I-lines remained more virulent than the N-lines to naïve mice, though not to immunized mice (Figure 1B). We discuss these two principal findings separately below.

### Immunity Selects for Higher Virulence

**Figure 2:**
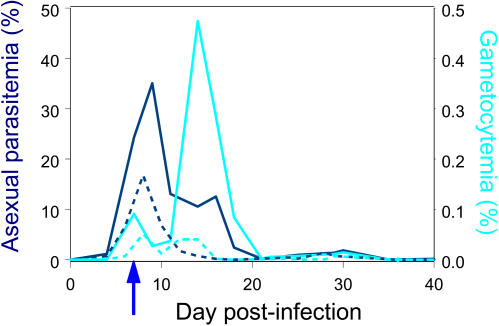
Effect of Immunization on Asexual Parasitemia and Gametocytemia Each curve represents the mean asexual parasitemia (dark blue) and gametocytemia (light blue) over all parasite lines (ancestral and selected) in naïve (solid lines; *n* = 47) and immunized mice (broken lines; *n =* 50) during the pretransmission evaluation phase. Immunization reduced asexual parasitemia and gametocytemia throughout the infection (*p* < 0.001 based on the log_10_ daily average taken over all days). The arrow indicates the day of transfer during the selection phase of the experiment.

The results suggest that immune selection on blood-stage parasites is more efficient at selecting virulent variants than is selection in naïve mice. Response to selection is a function of the amount of variation in the population and the proportion of the population that survives to produce offspring, i.e., the selection intensity. The higher selection response in the I-lines is unlikely to be due to greater variation on which selection could act because the parasite population size on the day of transfer in immunized mice was on average 2-fold smaller than in naïve mice (Figure 2). It is also unlikely to be due to lower host death in the I-lines as there were no line differences in mortality in naïve mice over the entire course of the experiment (10/223 naïve mice infected with N-lines versus 2/40 naïve mice infected with I-lines, *p* > 0.10 by 2-tailed Fisher's Exact test, zero mortality in immunized mice), and all but one of the deaths occurred after the day of transfer. The most likely explanation is that immunity generated more intense selection by killing a greater proportion of the parasite population up until the point of transfer (Figure 2). Winners of the race into the syringe on day 7 were those parasite variants that survived immune selection, and these parasites proceeded to cause more damage to their host later in the infection.

**Figure 3:**
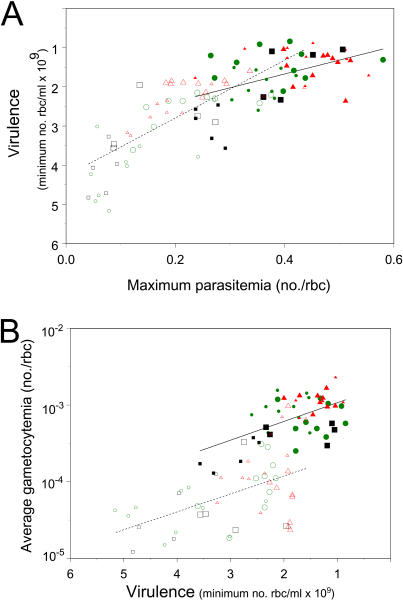
Relationships between Virulence, Asexual Multiplication, and Transmission Potential across Ancestral and Selection Lines Virulence, as measured by minimum red blood cell density, is plotted against maximum parasitemia in (A), and average daily gametocyte production (a measure of lifetime transmission potential) is plotted against virulence in (B). Data are all from pretransmission lines—ancestral and selected—measured in naïve (closed symbols; solid line) and immunized mice (open symbols; broken line). Regression analysis for both traits showed significant (*p* < 0.001) and similar (*p* > 0.05) slopes within both naïve and immunized mice, and significantly lower (*p* < 0.001) maximum parasitemia and gametocyte production in immunized than in naïve mice. When the two data points from naïve mice with values of above 3 × 10^9^ rbc/ml were excluded from the analyses, the slopes remained statistically similar (*p* > 0.05). Unselected ancestral populations, black squares; N-lines, green circles; I-lines, red triangles; avirulent unadapted ancestral population, small symbols; virulent preadapted ancestral population, large symbols.

But why would selection favor more virulent parasites? Our previous studies have consistently shown that peak parasite densities in the acute phase are positively correlated to the level of virulence that they generate (Mackinnon and Read 1999a, 1999b, 2003; Mackinnon et al. 2002; Ferguson et al. 2004). We therefore expected to find that the higher virulence in I-lines was accompanied by higher parasite densities, in which case we would deduce that immune selection had favored variants that were better able to outgrow immune defenses. While we found positive relationships between asexual multiplication and virulence across all the lines including the ancestral ones (Figure 3A), the I-lines and N-lines were statistically indistinguishable (*p* > 0.05) for (i) parasitemia on day 4, (ii) parasitemia on day 6 or 7, (iii) the increase in parasitemia from day 4 to day 6 or 7, and (iv) maximum parasitemia, with one exception: maximum parasitemia was significantly higher in I-lines than N-lines derived from unadapted ancestors when measured in immunized mice, and this only in one of the two replicate experiments (23% versus 6.9% parasitemia, *p* < 0.001). Thus, there is little evidence to suggest that the increased virulence was due to a higher asexual multiplication rate (or a lower death rate of asexuals) in those parasites that successfully made it into the syringe. Our data demonstrate that immunity acts as a powerful and upward inhost selective force on virulence, but the precise mechanism awaits further study.

There were positive relationships between virulence and lifetime transmission potential across all the lines (Figure 3B), consistent with our previous studies (reviewed in Mackinnon and Read 2004), but the differences between the I-lines and N-lines were not statistically significant (*p* > 0.05). Gametocyte densities are a good predictor of transmission probability in P. chabaudi and other *Plasmodium* species (Mackinnon and Read 2004), so these results demonstrate that the more virulent parasites evolved in semi-immune mice would transmit as successfully as the less virulent parasites evolved in naïve hosts. Thus, in the absence of a cost, virulent variants favored by within-host immune selection are expected to spread throughout an immunized host population.

### The Effects of Mosquito Transmission

Malaria parasites, like many microbes (Ebert 1998), are remarkable in their ability to rapidly adapt to changes in their host environment, and some of this is known to be due to phenotypic switching mechanisms in virulence-related phenotypes such as binding to host cells (Barnwell et al. 1983), red cell surface antigen expression (Brown and Brown 1965; Barnwell et al. 1983; David et al. 1983; Handunetti et al. 1987; Gilks et al. 1990), and red cell invasion pathways (Dolan et al. 1990). Some of these phenotype-based changes are transient, while others appear to be stable, i.e., maintained over sequential blood-stage passages. In our experiment, it is possible that the increases in virulence we observed following serial passage were at least partly due to altered gene expression rather than changes at the genome level. The public health consequences of this sort of change depend on whether the higher virulence is maintained during mosquito transmission, and upon transfer to hosts with different levels of immunity from those in which selection took place. We found that the I-lines were more virulent than the N-lines in both naïve and immunized hosts (see Figure 1A). However, after mosquito transmission, the I-lines remained more virulent than the N-lines, only in naïve hosts: the difference in immune hosts was negated by mosquito transmission (see Figure 1B). Possible reasons for this are discussed further below. For now, we note that the data are consistent with (though do not directly test) the prediction (Gandon et al. 2001) that enhancement of host immunity by anti-blood-stage vaccination will render malaria populations more dangerous to naïve hosts, at least in the short- to medium-term. Whether or not our long-term prediction (Gandon et al. 2001) that immunized populations will drive virulence to a higher level at evolutionary equilibrium proves true can be established only by monitoring vaccine-covered parasite populations in the field.

We observed a general reduction in virulence across all lines following mosquito transmission (see Figure 1), particularly when measured in immunized mice, and particularly in lines that had been selected under immune pressure, i.e., the I-lines, and in the CW-A ancestral line, which had been serially passaged on day 12 postinfection (PI). Many laboratory studies in malaria have shown that high or low virulence phenotypes accrued through serial passage can be maintained upon transmission through mosquitoes (James et al. 1936; Coatney et al. 1961; Alger et al. 1971; Walliker et al. 1976; Knowles and Walliker 1980; Walliker 1981; Barnwell et al. 1983), although occasional major losses (or gains) of virulence do occur (Alger et al. 1971; Walliker et al 1976; Knowles and Walliker 1980; Gilks et al. 1990). Mosquito transmission could play a significant role in virulence evolution that is driven by inhost selective processes (as distinct from the between-host selective processes underlying the vaccination hypothesis in Gandon et al. [2001]).

The mechanistic basis for the reduction in virulence following mosquito transmission remains to be determined. We offer the following speculations. It may be that the virulence reductions we and others have observed are due to stochastic loss of virulent variants during the population bottlenecking that occurs during mosquito transmission (the variability between lines in virulence loss during mosquito transmission favors this hypothesis). Alternatively, virulence reduction may be due to the deterministic forces of selection against virulent variants that have lost or reduced the ability to transmit through mosquitoes (Ebert 1998): the potential trade-off between virulence in the vertebrate host and production and infectivity of sporozoites in the mosquito has not yet been explored. A further possibility is that the virulence reductions observed following mosquito transmission are due to the systematic resetting during meiosis of the expression of genes that have been switched on or up-regulated during asexual serial passage. For example, it is known that mosquito transmission induces the expression of a different set of the clonally variant (i.e., phenotypically switching) surface antigens from those expressed at the time of ingestion by the mosquito (McLean et al. 1987; Peters et al. 2002). It is possible that the variants that appear early in the infection, either because of some genetically programmed ordering of expression or because of higher intrinsic switching rates, are recognized by the immune system in a preimmunized host, thus giving the late-appearing variants a selective advantage. Our data are consistent with this idea, since mosquito transmission eliminated the difference between the I-lines and N-lines in immunized mice but not in naïve mice, suggesting that part of the virulence advantage in immunized hosts was due to novelty in the clonally variant surface antigens. Finally, an interesting possibility is that it is loss of diversity per se during mosquito transmission (either at the genetic level or at the phenotypic expression level) that causes a reduction in virulence by limiting the invading parasites' ability to evade immune defenses: our data are also consistent with this hypothesis.

Any of these mechanisms could explain the loss of virulence during mosquito transmission, but none are sufficient to explain why the I-lines were more virulent than the N-lines in naïve mice both before and after mosquito transmission. Thus, more than one distinct underlying mechanism probably explains the virulence differences observed here, such as differences in intrinsic virulence properties and differences in levels of antigenic diversity within the lines. Identifying the mechanisms, any links between them, and their relative roles in determining parasite survival in naïve versus immunized hosts are of key importance in understanding virulence evolution and immunoepidemiology of malaria in the field.

### Other Serial Passage Studies in Malaria

To what extent do our observations accord with previous work on serial passage of malaria in immune-modified environments? Results from other studies are difficult to interpret as none maintained control lines for selection (i.e., lines that were passaged in the nonmanipulated immune environment), most had no replication of lines within selection treatment, and some used just a single selection step. Nevertheless, some tentative conclusions may be drawn. Comparisons of selected and ancestral parasites have been made after three different forms of immune manipulation: (i) down-regulation of immunity by removal of the spleen prior to infection, (ii) up-regulation of immunity by transfer of immune serum at the beginning of infection, and (iii) up-regulation of immunity by infection, sometimes with subcurative drug treatment in order to establish a chronic infection. In the first two, parasites were selected from the primary wave of parasitemia, as in our experiment, whereas in the third, selected parasites were isolated from relapses much later in the infection (40–150 d PI). Parasite lines passaged through splenectomized hosts often lose the ability to bind to host endothelial cells (cytoadherence) in the microvasculature of the deep tissues and therefore the ability to avoid being passaged through the spleen (Garnham 1970), the primary site of immune-mediated clearance (Wyler 1983). This loss of binding is often accompanied by a loss of ability to express (Barnwell et al. 1983; Handunetti et al. 1987; Gilks et al. 1990)—or a major alteration in the level of expression of (David et al. 1983; Fandeur et al. 1995)—the highly variable and clonally variant switching parasite antigens on the surface of the red cell known to be important for the maintenance of long-term chronic infections (Brown and Brown 1965). In P. falciparum at least (David et al. 1983; Hommel et al. 1983), this coincident change in the two properties is because both phenotypes are mediated by the same parasite molecule, denoted PfEMP1 (Baruch et al. 1995; Smith et al. 1995; Su et al. 1995). Importantly, in two of three studies, the line of parasites that lost cytoadherence and/or surface antigen expression had much-reduced virulence to spleen-intact naïve hosts compared to their ancestral lines (Barnwell et al. 1983; Langreth and Peterson 1985; Gilks et al. 1990). If our immunization procedure was priming the spleen for effective parasite clearance, our results are consistent with these findings.

However, the second form of immune selection—passage of acute-phase parasites from hosts injected with antiserum at the beginning of the infection—yielded parasites with lower virulence to naïve mice than their ancestors in one study (Wellde and Diggs 1978), although it had no impact on virulence in two other studies (see Briggs and Wellde 1969). The third type of immune selection—isolation of parasites from relapses late in the infection—has generated parasites with virulence to naïve hosts that is lower than (Cox 1962), higher than (Sergent and Poncet 1955), or similar to (Cox 1959) that of their ancestors. In all these studies, which involved only single passages, selected parasites were more virulent than their ancestors to immunized hosts, suggesting that the selected parasites were predominantly of a novel antigenic type (a fact that has sometimes been demonstrated; Voller and Rossan 1969). Whether antigenic novelty is traded off against multiplication rate or virulence among the repertoire of variants expressed during a single infection—as has also been suggested from field population studies (Bull et al. 1999)—is an interesting question that deserves more attention. However, in our study, in which we focused on the longer-term and more natural environment of hosts preimmunized with a heterogeneous parasite population, the higher virulence of the I-lines compared to the N-lines in both naïve and immunized mice leads us to deduce that selection associated with virulence overrides selection for immune evasion alone.

### Conclusion

Our data demonstrate that host immunity can increase the potency of inhost selection for higher virulence in malaria. Whether our results generalize to other immunization protocols, parasite clones, parasite species, host genotypes, repeated mosquito passage, and so on requires extensive further experimentation. But, coupled with the malaria parasite's famous ability to rapidly adapt to novel conditions in the laboratory (see above) and to variant-specific vaccine pressure (Genton et al. 2002) and drugs (Peters 1987) in the field, these results urge the continuous monitoring of virulence of parasite populations if asexual-stage malaria vaccines become widely used. And for other microparasites (bacteria, viruses, and protozoa) that rely on rapid multiplication within the host for successful transmission, similar concerns might apply.

## Materials and Methods

### 

#### Selection phase.

Starting from two separate ancestral lines derived from clone CW (see below), five parasite lines (“sublines”) from each ancestral line were repeatedly passaged in mice (female C57Bl/6J, 7–10 wk old) that were naïve to malaria infection (N-lines), and five from each ancestral line were passaged in immunized mice (I-lines, see below), forming 20 lines (“sublines”) in total. Passages involved the syringe transfer to a fresh mouse of 0.1 ml of diluted blood containing 5 × 10^5^ parasites from a donor mouse that had been infected 7 d previously. Day 7 PI is during the period of rapid population growth, and is about 2 d prior to peak parasitemia, after which population size rapidly declines (see Figure 2). Parasite lines under the same selection regime (i.e., passage in immune versus naïve mice) were not mixed at each transfer, thus yielding five independent replicate sublines in each of the four selection treatment–ancestral line groups.

Immunization was by infection with 10^4^ parasites of a different clone (denoted ER), followed by drug cure with 10 mg/kg of mefloquine for 4 d starting on day 5 PI. Naïve mice were injected with parasite-free media but were not drug treated. Re-infection took place on average 3 wk after the end of drug treatment (range 1.5–5 wk): as the half-life of mefloquine in mice is reported to be 18 h (Peters 1987), the residual amount in the blood by this stage was expected to be very low. The same deep-frozen stock of ER was used each generation. ER is genetically distinct from CW at marker loci (data not shown) and was originally isolated from different hosts. Before use in this experiment, ER had undergone two passages since mosquito transmission and more than 20 passages prior to that. No recrudescent infections in immunized mice were detected prior to challenge. In generations 10 and 11, all lines were passaged through naïve mice.

The serial passage experiments in this study were replicated using two different starting populations (ancestral lines)—one avirulent (CW-0) and one virulent (CW-A). CW-0 had been cloned by serial dilution from an isolate obtained from its natural host, the thicket rat, *Thamnomys rutilans,* and then blood passaged every 12 d for a total of 12 passages to produce the CW-A line. During these passages, CW-A was subjected to selection for low virulence on the basis of how much weight loss it caused to mice. Despite this selection, however, CW-A increased in virulence relative to CW-0 during these passages (Mackinnon and Read 1999b). Prior to use in the current experiments, both CW-0 and CW-A underwent four further serial passages in naïve mice, and were not recloned.

All the lines, including the ancestral lines, were transmitted once through Anopheles stephensi mosquitoes by allowing 50–100 mosquitoes aged 2–5 d to take a blood meal for 20–30 min on an anaesthetized gametocytemic mouse that had been inoculated 6–10 d previously, i.e., prior to the peak of infection. Then, 11–12 d later these mosquitoes—typically 10–20 of them infected as assessed by random surveys of oocyst prevalence—were allowed to feed back onto anaesthetized naïve mice. After 7–10 d, the blood from these sporozoite-infected mice was harvested and stored in liquid nitrogen. These aliquots were used to initiate blood infections in naïve mice that were then used as donors of asexual parasites to mice involved in the posttransmission experiments. As the lines were transmitted through mosquitoes noncontemporaneously, and involved typically one mouse per subline, comparisons among the lines for infectivity to mosquitoes were not made during these transmission exercises.

#### Evaluation phase.

After 18 passages, the pretransmission lines were evaluated in two replicate experimental blocks in naïve (generations 19 and 21) and immunized mice (generations 20 and 22). Ancestral lines were only evaluated in generations 21 and 22. This set of trials was denoted the “pretransmission experiments.” In a separate set of experiments, the “posttransmission experiments,” the mosquito-transmitted lines were compared with each other, as well as with the nontransmitted ancestral lines in two replicate experimental blocks in both naïve (generations 23 and 24) and immunized mice (generations 25 and 26). In both these experiments, across both blocks, ten mice were used for each of the four selection groups (two per subline), and five mice were used per ancestral line. Red blood cell density was measured every 1 or 2 d until day 18 PI by flow cytometry (Coulter Electronics, Luton, United Kingdom), and the minimum density reached was taken as a measure of virulence. Liveweight of the mouse was also recorded every 1–2 d. During the pretransmission experiments (generations 19–22), parasitemia and gametocytemia (proportions of red blood cells infected with asexual parasites and gametocytes, respectively) were evaluated from Giemsa-stained thin blood smears every 2 d from day 4 PI until day 18 PI, and then four more times until day 43 PI. Total lifetime transmission potential was measured as the average gametocytemia throughout the infection from day 4 to day 18 PI.

#### Analysis.

Statistical analyses were performed separately for the pretransmission and posttransmission experiments as these were carried out at different times. The virulence measure used for the final analysis was minimum red blood cell density, though other measures of virulence were also analyzed (unpublished data). Since selection treatment was replicated on sublines, thus making subline the independent experimental unit, the means of mice within sublines were first calculated. These were then analyzed for the effects of immune environment on selection response by fitting a linear model to these data with factors for selection line (with three levels for nontransmitted ancestral lines, N-lines, and I-lines in the case of the pretransmission experiments, and four levels for the transmitted versions of these three lines plus the nontransmitted ancestral lines in the case of the posttransmission experiments), ancestral population (CW-0, CW-A), and an interaction between these two factors. Thus, statistical tests of differences between the selection lines and other factors in the model were made using t-tests, with the variance for subline means as the residual. An alternative model fitted to data on individual mice (rather than means of sublines) that incorporated subline as a random effect was found to be unsatisfactory because in some treatment groups, the model did not converge and estimates of the subline variance were highly variable between groups. To determine the effects of mosquito transmission on the line differences in virulence, a further analysis was performed on the combined data from the pretransmission and posttransmission experiments fitting a fixed effect factor of line-within-experiment in the statistical model (seven levels—three lines for the pretransmission experiment and four for the posttransmission experiment). These analyses were carried out separately for each of the four immune-treatment-by-ancestral-line groups. Since the pretransmission ancestral line was included in both the pretransmission and posttransmission experiments, the effect of mosquito transmission (and its standard error) on the N-lines and I-lines, which was not measured directly (i.e., in a single experiment), could be estimated by reference to this line. For example, the effect of mosquito transmission in the N-lines was estimated from the difference between the N-lines and their pretransmission ancestral line in the pretransmission experiment minus the analogous contrast in the posttransmission experiment. This was done using the method of linear contrasts provided for in the SAS GLM procedure (SAS 1990). The effect of mosquito transmission on the difference between the I-lines and N-lines was similarly calculated but without reference to the pretransmission ancestral line. The effect of mosquito transmission on the ancestral lines was estimated from the direct comparison available from only the posttransmission experiment data.

## Abbreviations

I-line: immune-selected line
N-line: naïve-selected line

## References

[pbio-0020230-Alger1] Alger NE, Branton M, Harant J, Silverman PH (1971). Plasmodium berghei NK65 in the inbred A/J mouse: Variations in virulence in P. berghei demes. J Protozool.

[pbio-0020230-Antia1] Antia R, Levin BR, May RM (1994). Within-host population dynamics and the evolution and maintenance of microparasite virulence. Am Nat.

[pbio-0020230-Barnwell1] Barnwell JW, Howard RJ, Miller LH, Evered D, Whelan J (1983). Influence of the spleen on the expression of surface antigens on parasitized erythrocytes. CIBA Foundation symposium on malaria and the red cell.

[pbio-0020230-Baruch1] Baruch DI, Pasloske BL, Singh HB, Bi X, Ma XC (1995). Cloning the P. falciparum gene encoding pfEMP1, a malarial variant antigen and adherence receptor on the surface of parasitized human erythrocytes. Cell.

[pbio-0020230-Best1] Best SM, Kerr PJ (2000). Coevolution of host and virus: The pathogenesis of virulent and attenuated strains of myxoma virus in resistant and susceptible European rabbits. Virology.

[pbio-0020230-Briggs1] Briggs NT, Wellde BT (1969). Some characteristics of Plasmodium berghei “relapsing” in immunized mice. Mil Med.

[pbio-0020230-Brown1] Brown KN, Brown IN (1965). Immunity to malaria: Antigenic variation in chronic infections of Plasmodium knowlesi. Nature.

[pbio-0020230-Brown2] Brown SP, Hochberg ME, Grenfell BT (2002). Does multiple infection select for raised virulence?. Trends Microbiol.

[pbio-0020230-Bull1] Bull PC, Lowe BS, Kortok M, Marsh K (1999). Antibody recognition of Plasmodium falciparum erythrocyte surface antigens in Kenya: Evidence for rare and prevalent variants. Infect Immun.

[pbio-0020230-Chao1] Chao L, Hanley KA, Burch CL, Dahlberg C, Turner PE (2000). Kin selection and parasite evolution: Higher and lower virulence with hard and soft selection. Q Rev Biol.

[pbio-0020230-Coatney1] Coatney GR, Elder HA, Contacos PG, Getz ME, Greenland R (1961). Transmission of the M strain of Plasmodium cynomolgi to man. Am J Trop Med Hyg.

[pbio-0020230-Cox1] Cox HW (1959). A study of the relapse Plasmodium berghei infections isolated from white mice. J Immunol.

[pbio-0020230-Cox2] Cox HW (1962). The behavior of Plasmodium berghei strains isolated from relapsed infections of white mice. J Protozool.

[pbio-0020230-David1] David PH, Hommel M, Miller LH, Udeinya IJ, Oligino LD (1983). Parasite sequestration in Plasmodium falciparum malaria: Spleen and antibody modulation of cytoadherence of infected erythrocytes. Proc Natl Acad Sci U S A.

[pbio-0020230-Dieckmann1] Dieckmann U, Metz H, Sabelis MW, Sigmund K (2002). Virulence management: The adaptive dynamics of pathogen-host interactions.

[pbio-0020230-Dolan1] Dolan SA, Miller LH, Wellems TE (1990). Evidence for a switching mechanism in the invasion of erythrocytes by Plasmodium falciparum. J Clin Invest.

[pbio-0020230-Ebert1] Ebert D (1998). Experimental evolution of parasites. Science.

[pbio-0020230-Fandeur1] Fandeur T, Le Scanf C, Bonnemains B, Slomianny C, Mercereau-Puijalon O (1995). Immune pressure selects for Plasmodium falciparum parasites presenting distinct red blood cell surface antigens and inducing strain-specific protection in Saimiri sciureus monkeys. J Exp Med.

[pbio-0020230-Fenner1] Fenner F, Ratcliffe RN (1965). Myxomatosis.

[pbio-0020230-Ferguson1] Ferguson HM, Mackinnon MJ, Chan BHK, Read AF (2004). Mosquito mortality and the evolution of malaria virulence. Evolution.

[pbio-0020230-Frank1] Frank SA (1996). Models of parasite virulence. Q Rev Biol.

[pbio-0020230-Gandon1] Gandon S, Mackinnon MJ, Nee S, Read AF (2001). Imperfect vaccines and the evolution of parasite virulence. Nature.

[pbio-0020230-Garnham1] Garnham PCC (1970). The role of the spleen in protozoal infections with special reference to splenectomy. Acta Trop.

[pbio-0020230-Genton1] Genton B, Betuela I, Felger I, Al-Yaman F, Anders RF (2002). A recombinant blood-stage malaria vaccine reduces Plasmodium falciparum density and exerts selective pressure on parasite populations in a phase 1–2b trial in Papua New Guinea. J Infect Dis.

[pbio-0020230-Gilks1] Gilks CF, Walliker D, Newbold CI (1990). Relationships between sequestration, antigenic variation and chronic parasitism in *Plasmodium chabaudi chabaudi* A rodent malaria model. Parasite Immunol.

[pbio-0020230-Handunetti1] Handunetti SM, Mendis KN, David PH (1987). Antigenic variation of cloned Plasmodium fragile in its natural host Macaca sinica. J Exp Med.

[pbio-0020230-Hommel1] Hommel M, David PH, Oligino LD (1983). Surface alterations of erythrocytes in Plasmodium falciparum malaria: Antigenic variation, antigenic diversity and the role of the spleen. J Exp Med.

[pbio-0020230-James1] James SP, Nicol WD, Shute PG (1936). Clinical and parasitological observations on induced malaria. Proc R Soc Med.

[pbio-0020230-Knowles1] Knowles G, Walliker D (1980). Variable expression of virulence in the rodent malaria parasite *Plasmodium yoelii yoelii*. Parasitology.

[pbio-0020230-Koella1] Koella JC, Antia R (1995). Optimal pattern of replication and transmission for parasites with two stages in their life-cycle. Theor Pop Biol.

[pbio-0020230-Langreth1] Langreth SG, Peterson E (1985). Pathogenicity, stability and immunogenicity of a knobless clone of Plasmodium falciparum in Colombian owl monkeys. Infect Immun.

[pbio-0020230-Mackinnon1] Mackinnon MJ, Read AF (1999a). Genetic relationships between parasite virulence and transmission in the rodent malaria Plasmodium chabaudi. Evolution.

[pbio-0020230-Mackinnon2] Mackinnon MJ, Read AF (1999b). Selection for high and low virulence in the malaria parasite Plasmodium chabaudi. Proc R Soc Lond B Biol Sci.

[pbio-0020230-Mackinnon3] Mackinnon MJ, Read AF (2003). Effects of immunity on relationships between growth rate, virulence and transmission in semi-immune hosts. Parasitology.

[pbio-0020230-Mackinnon4] Mackinnon MJ, Read AF (2004). Virulence in malaria: An evolutionary viewpoint. Philos Trans R Soc Lond B Biol Sci.

[pbio-0020230-Mackinnon5] Mackinnon MJ, Gaffney DJ, Read AF (2002). Virulence in malaria parasites: Host genotype by parasite genotype interactions. Infect Genet Evol.

[pbio-0020230-McLean1] McLean SA, Phillips RS, Pearson CD, Walliker D (1987). The effect of mosquito transmission of antigenic variants of Plasmodium chabaudi. Parasitology.

[pbio-0020230-Nowak1] Nowak MA, May RM (1994). Superinfection and the evolution of parasite virulence. Proc R Soc Lond B Biol Sci.

[pbio-0020230-Peters1] Peters W (1987). Chemotherapy and drug resistance in malaria.

[pbio-0020230-Peters2] Peters J, Fowler E, Gatton M, Chen N, Saul A (2002). High diversity and rapid changeover of expressed *var* genes during the acute phase of Plasmodium falciparum infections in human volunteers. Proc Natl Acad Sci U S A.

[pbio-0020230-Read1] Read AF, Taylor LH (2001). The ecology of genetically diverse infections. Science.

[pbio-0020230-SAS1] [SAS] SAS Institute (1990). SAS/STAT user's guide, version 6.0.

[pbio-0020230-Sergent1] Sergent E, Poncet A (1955). Étude expérimentale du paludisme des rongeurs à Plasmodium berghei. II. Stade d'infection latente métacritique. Arch Inst Pasteur Alger.

[pbio-0020230-Smith1] Smith JD, Chitnis CE, Craig AG, Roberts DJ, Hudson-Taylor DE (1995). Switches in expression of *Plasmodium falciparum var* genes correlate with changes in antigenic and cytoadherent phenotypes of infected erythrocytes. Cell.

[pbio-0020230-Su1] Su X, Heatwole VM, Wertheimer SP, Guinet F, Herrfeldt JA (1995). The large diverse gene family *var* encodes proteins involved in cytoadherence and antigenic variation of *Plasmodium falciparum–*infected erythrocytes. Cell.

[pbio-0020230-Van1] Van Baalen M, Sabelis MW (1995). The dynamics of multiple infection and the evolution of virulence. Am Nat.

[pbio-0020230-Voller1] Voller A, Rossan RN (1969). Immunological studies with simian malarias. I. Antigenic variants of *Plasmodium cynomolgi bastianellii*. Trans R Soc Trop Med Hyg.

[pbio-0020230-Walliker1] Walliker D, Canning EU (1981). The genetics of virulence in Plasmodium yoelii. Parasitological topics.

[pbio-0020230-Walliker2] Walliker D, Sanderson A, Yoeli M, Harant J, Hargreaves B (1976). A genetic investigation of virulence in a rodent malaria parasite. Parasitology.

[pbio-0020230-Wellde1] Wellde BT, Diggs CL (1978). Plasmodium berghei: Biological variation in immune serum–treated mice. Exp Parasitol.

[pbio-0020230-Wyler1] Wyler DJ, Evered D, Whelan J (1983). The spleen in malaria. Ciba Foundation symposium on malaria and the red cell.

